# Eluxadoline-Loaded Eudragit Nanoparticles for Irritable Bowel Syndrome with Diarrhea: Formulation, Optimization Using Box–Behnken Design, and Anti-Diarrheal Activity

**DOI:** 10.3390/pharmaceutics15051460

**Published:** 2023-05-10

**Authors:** Md. Khalid Anwer, Mohammed Muqtader Ahmed, Mohammed F. Aldawsari, Muzaffar Iqbal, Gamal A. Soliman, Ibrahim A. Aljuffali

**Affiliations:** 1 Department of Pharmaceutics, College of Pharmacy, Prince Sattam Bin Abdulaziz University, Al-Kharj 11942, Saudi Arabia; 2 Department of Pharmaceutical Chemistry, College of Pharmacy, King Saud University, Riyadh 11451, Saudi Arabia; 3 Bioavailability Laboratory, College of Pharmacy, King Saud University, Riyadh 11451, Saudi Arabia; 4 Department of Pharmacology and Toxicology, College of Pharmacy, Prince Sattam Bin Abdulaziz University, Al-Kharj 11942, Saudi Arabia; 5 Department of Pharmacology, College of Veterinary Medicine, Cairo University, Giza 12613, Egypt; 6 Department of Pharmaceutics, College of Pharmacy, King Saud University, Riyadh 11451, Saudi Arabia

**Keywords:** eluxadoline, eudragit, optimization, dissolution, defecation frequency, disease activity index

## Abstract

Eluxadoline (ELD), a recently approved drug, exhibits potential therapeutic effects in the management and treatment of IBS-D. However, its applications have been limited due to poor aqueous solubility, leading to a low dissolution rate and oral bioavailability. The current study’s goals are to prepare ELD-loaded eudragit (EG) nanoparticles (ENPs) and to investigate the anti-diarrheal activity on rats. The prepared ELD-loaded EG-NPs (ENP1-ENP14) were optimized with the help of Box–Behnken Design Expert software. The developed formulation (ENP2) was optimized based on the particle size (286 ± 3.67 nm), PDI (0.263 ± 0.01), and zeta potential (31.8 ± 3.18 mV). The optimized formulation (ENP2) exhibited a sustained release behavior with maximum drug release and followed the Higuchi model. The chronic restraint stress (CRS) was successfully used to develop the IBS-D rat model, which led to increased defecation frequency. The in vivo studies revealed a significant reduction in defecation frequency and disease activity index by ENP2 compared with pure ELD. Thus, the results demonstrated that the developed eudragit-based polymeric nanoparticles can act as a potential approach for the effective delivery of eluxadoline through oral administration for irritable bowel syndrome diarrhea treatment.

## 1. Introduction

Irritable bowel syndrome (IBS) is one of the common types of gastrointestinal disorders, having a global incidence of 11–15% [1,2]. The most common symptoms of IBS include systematic pain in the abdominal region related to the alterations in the frequency of stools and are categorized depending upon the major stool patterns: IBS-diarrhea (IBS-D), IBS-constipation, both mixed, or uncategorized. Amongst all these, the most common category is IBS, which comprises nearly 45–50% of all cases of IBS [3]. Studies have reported limited therapeutic strategies for the effective management of IBS-D. Conventional therapeutic strategies involve anti-diarrheals and modifications in the diet, as well as lifestyle, of the patient. A negative impact is observed in the quality of life of the patients due to IBS-D, surpassing the quality-of-life deteriorations observed in asthmatic, gastrointestinal reflux diseases [4,5], and even IBS constipation [6]. Numerous comorbid circumstances have been related with IBS, such as fibromyalgia, migraine headaches, interstitial cystitis, major depression, anxiety, and others [7]. IBS-D executes a considerable financial burden on society and financiers due to augmented healthcare uses [8,9,10].

Loperamide (LPM) is one of the most used therapeutics for the effective management of IBS-D. LPM is a peripherally acting m-opioid receptor (m-OR) agonist that pharmacologically reduces the gut motility and enhances fluid re-absorption [11,12]. However, LPM is not directed for long-term usage and does not regulate abdominal pain or swelling [13,14,15]. Thus, there is substantial necessity for new and effective therapies with promising safety profiles exhibiting potential effects of long-term reprieve from symptoms of IBS-D patients.

**Figure 1:**
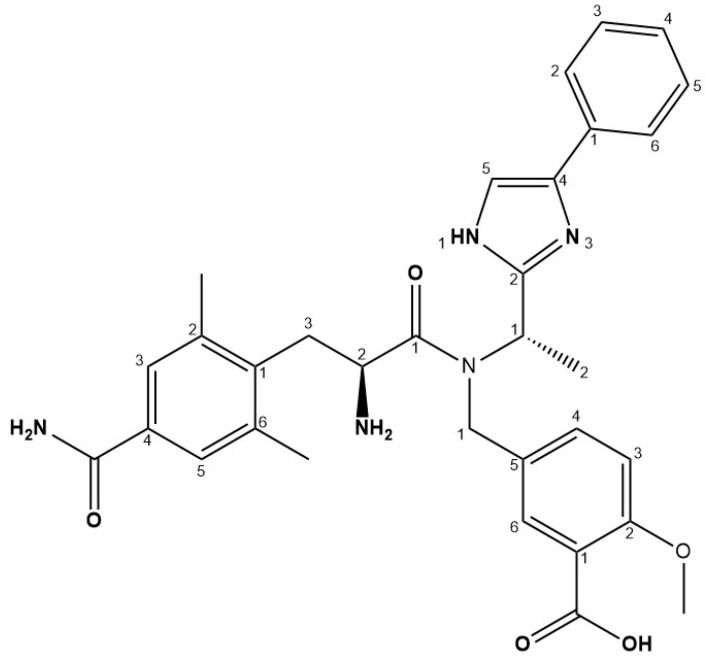
Chemical structure of eluxadoline.

Eluxadoline (ELD) is local-acting and exhibits diverse pharmacological effects, including m-OR and kappa opioid receptor agonists and delta opioid receptor antagonists. It exerts negligible oral bioavailability yet is officially approved by the United States Food and Drug Administration for IBS-D treatment [16]. Due to its collective pharmacological properties, ELD (Figure 1) significantly decreases the gut motility and reduces the probabilities of drug-induced constipation issues. Studies, including the animal models, have demonstrated that the combinatorial dosage of a d-opioid receptor antagonist and an l-opioid receptor agonist significantly improves its effects on intuitive sensations, while reduces the possibility of constipation [17,18]. These exclusive properties differentiate ELD from peripherally acting m-OR agonists such as LPR [18]. ELD is commercially available in tablet dosage form (75 and 100 mg) for treating IBS-D, but other dosage forms, including capsules and liquid systems for pediatric usage, are commercially unavailable [19].

More importantly, ELD shows poor bioavailability, primarily due to poor aqueous solubility, restricted absorption, and first-pass metabolism, and thus its frequent use is limited [20]. Thus, a novel and targeted therapeutic strategy is necessary to improve the solubility, permeability, and oral bioavailability of ELD, with reduced or negligible toxicity profiles, for the effective management of IBS-D. Nanotechnology-derived therapeutic strategies, including polymeric nanocarriers, lipid-based nanoparticles, metallic nanoparticles, and others have shown immense potential for delivering therapeutics to targeted disease sites with controlled release behavior, particularly for delivering drugs with low oral bioavailability [21].

Polymeric nanocarrier systems have shown immense potential and have attracted significant interest over recent years owing to their diverse characteristics [22,23]. Several drugs have been successfully delivered using the nanoencapsulation of drugs, which has been shown to increase solubility, protect against toxicity, increase pharmacological activity and stability, provide sustained release, provide physical protection, and protect against chemical degradation [23,24,25,26]. Amongst various polymers, eudragit is one of the most used polymers for the fabrication of drug-loaded polymeric systems. Eudragit exhibits protective effects against the environmental moisture, masks the smells/flavors, and also shows versatility, subsequently due to the choice of a precise type as per the required delivery system, i.e., sustained, immediate, or controlled. Moreover, it is also well-defined as a pH-sensitive polymer due to its solubilizing abilities at varying pH values [27,28].

The aim of the current study was to enhance the oral bioavailability of ELD through loading into eudragit nanoparticles and to investigate the ability of ELD-loaded EG-NPs’ (ENPs) effective treatment of IBS-D. The optimization of developed formulations was performed using Box–Behnken Design Expert software, considering independent variables such as weight of eudragit polymer, PVA (%*w*/*v*), and sonication time and dependent variables such as particle size, polydispersity index, and zeta potential. The optimized ELD-loaded EG-NPs (ENP2) were finally evaluated for different pharmaceutical attributes and their ability to improve the oral efficacy against irritable bowel syndrome with diarrhea.

## 2. Materials and Methods

### 2.1. Materials

Eluxadoline (ELD) was procured from Mesochem Technology Co., Ltd., Beijing, China. The poly (vinyl alcohol) and eudragit RS100 were purchased from Sigma-Aldrich (St. Louis, MO, USA). Analytical grade chemicals and reagents were employed throughout the experiment.

### 2.2. Box–Behnken Design (BBD) Optimization

A Box–Behnken response surface approach experimental design (Design-Expert^®^ Software Version 13) was used to optimize the eluxadoline-loaded eudragit nanoparticles (ENPs) (3 factors, 3 levels). The independent variables selected were: the weight of the eudragit polymer (X1), %*w*/*v*, PVA (X2), and sonication time (X3), with their high, medium, and low levels for the preparation of 14 formulations, as shown in Table 1. Particle size (Y1), polydispersity index (Y2), and zeta potential (Y3) were the responses that were examined. Additionally, 3D response surface graphs were plotted to show how the specified factors affected the responses that were measured.

### 2.3. Preparation of Eluxodoline-Loaded Eudragit Nanoparticles (ENPs)

ELD-loaded EG-NPs (ENPs) were prepared by the emulsion solvent diffusion method, using different proportions of EG (polymer) and PVA (stabilizer). The disperse phase consisted of ELD and EG in 5 mL of dichloromethane, which was further added in the PVA aqueous phase (10 mL) using a probe sonicator (ultrasonic processor, Fisher scientific, Waltham, MA, USA) at 65% W with 5 s on/off for 3 to 9 min. Thereafter, continuous stirring took place for 24 h at 1000 rpm using a magnetic stirrer. The prepared nanoparticles were collected by filtration and freeze dried (Millirock Technology, Kingston, NY, USA) and finally packed for further evaluations in a sealed vial.

### 2.4. Measurement of Particle Size, PDI, and Zeta Potential

The average particle size and PDI of all the developed formulations (ENP1-ENP14) were estimated using a Malvern zetasizer (ZEN-3600, Malvern Instruments Ltd., Worcestershire, WR14 1XZ, UK) at 25 ± 2 °C. The colloidal suspension of ENPs was diluted with deionized water (200 times) with a refractive index of 1.33 and a dielectric constant of 78.5; an angle of angle of measurement was set at 90° to incident laser light, ultrasonicated for 10 min, then the diluted sample (1.5 mL) was kept in the sample holder of the instrument in a disposable plastic cuvette, after which the particle size and PDI were measured three times. The zeta potential (ZP) of ENPs was measured following the same procedure, except using a glass electrode cuvette in the place of the glass cuvette [29]. The data were analyzed using a software DTS V–4.1 (Malvern, UK) equipped with the system; the measurements were performed in triplicate.

### 2.5. Drug Encapsulation

The drug encapsulation (%DE) in ELD-loaded EG-NPs (ENP2) was determined indirectly by estimating the supernatant after centrifugation at 15,000 rpm for 10 min. The drug in the filtrate was then quantified using the HPLC technique [30]. The ratio between the amount of drug encapsulated in the nanoparticles and the amount added to prepare NPs was used to calculate the %DE. The experiment was carried out three times.

$$\%DE=\frac{ELD\;added\;in\;NPs-ELD\;in\;supernatant}{ELD\;added\;in\;NPs}$$

### 2.6. DSC Analysis

DSC spectra of pure ELD, optimized ELD-loaded EG NPs (ENP2), eudragit (EG), and PVA were taken by a DSC thermal analyzer using Scinco N650 (made in Korea, Scinco, Seoul, South Korea). The sample (5 mg) was cramped into a hemispherical aluminum pan and placed beside a reference (empty pan) in the sample holder, supplied with nitrogen (20 mL/min), and heated at a rate of 20 °C in the temperature range of 50–300 °C.

### 2.7. FTIR Studies

FTIR studies assisted in estimating the probability of the interaction of the drug with the excipients. Initially, a blank sample of potassium bromide (KBr) was run to eliminate the background errors. Further, pure ELD, EG, PVA, and the optimized formulation (ENP2) were mixed separately with KBr, physically compressed to form a transparent film, and analyzed within a wavelength range of 4000–400 cm^−1^ (Jasco 4600 Mid-IR FTIR spectrometer, Jasco, Tokyo, Japan).

### 2.8. In Vitro Drug Release Studies

In vitro drug release studies were performed to analyze the drug release behavior and the release mechanism of the optimized formulation. The analysis of pure ELD and the optimized formulation (ENP2) were determined using the dialysis membrane method. The pure ELD and ENP2 (5 mL) were loaded into the dialysis bag (Spectra/Por^®^ Standard RC Tubing, MWCO 12 KDa) and tied at both ends. Then, the bags were dipped into a beaker containing a dissolution medium of pH 1.2 and pH 6.8 (200 mL), maintained at a temperature of 37 ± 2 °C, and kept under stirring at 100 rpm. At pre-estimated time intervals, 0.5 mL aliquots were withdrawn from each beaker and the sink condition was maintained by adding fresh media [31]. Furthermore, the aliquots were analyzed for drug content by the HPLC method and the percent drug release was calculated and plotted against the time. [30]. All the studies were performed in triplicate (n = 3). Additionally, the drug release mechanism of ELD from the optimized formulation at pH (1.2 and 6.8) was estimated by fitting the drug release results into different mathematical modeling, including zero order, first order, Higuchi, and Korsmeyer–Peppas kinetics models [32,33].

### 2.9. SEM Studies

The surface morphology of optimized NPs (ENP2) was visualized using SEM (Zeiss EVO LS10, Cambridge, UK) by using the gold sputter method. The imaging was performed after the sample was coated with gold and put in a stub.

### 2.10. Effect on Irritable Bowel Syndrome in Rats

#### 2.10.1. Animals

A total of 30 Wistar albino male rats weighing between 110 and 120 g were obtained from the lab animal unit, College of Pharmacy, University of Prince Sattam bin Abdulaziz. The animals were maintained in ventilated cages (Rat IVC Blue Line, Techniplast, Buguggiate VA, Italy) in controlled environmental conditions (25 ± 1 °C and 12 h/12 h light/dark cycle). All rats were fed a standard rat pellet and water ad libitum.

#### 2.10.2. Induction of Irritable Bowel Syndrome (IBS) Model

In this study, the IBS rat model was induced by chronic restraint stress (CRS) as described by Lu et al. [34]. All rats were randomly divided into two groups (24 rats in the model group and 6 rats in the control group) after 7 days of adaptation. The rats in the model group were subjected to CRS using an elastic bandage to restrict the movement of the upper body and forelimbs and then anesthetized with ether. Their fore shoulders, upper forelimbs, and thoracic trunk were wrapped in elastic bandage for 2 h each day for 14 days to produce a steady and consistent amount of stimulation to restrict but not prevent movement. The control animals were anesthetized with ether but not restrained.

##### Grouping and Administration

The rats were divided into five groups (n = 6) as follows:

1. Normal control group (NC): normal rats received saline (1 mL/kg).
2. IBS control group (IBS-C): CRS rats received saline (1 mL/kg).
3. Reference group (REF): CRS rats received loperamide (LRD) at 10 mg/kg.
4. Pure drug group (ELD-std): CRS rats received (20 mg/kg).
5. Formulation group (ENP2): CRS rats received (20 mg/kg).

Treatments were administered orally, started 6 h after IBS induction, and continued for 14 consecutive days. Body weights were recorded daily.

#### 2.10.3. Evaluation of Fecal Parameters

After 14 days of treatment, stool frequency and the wet weight of fecal pellets were recorded per rat over 12 h. The stool frequency of each rat was measured at 2 h intervals for 12 h (e.g., 0–2 h, 2–4 h, 4–6 h, etc.). The wet fecal pellets were weighed, desiccated in an oven (50 °C, 6 h), and weighed again (dry weight in mg). The ratio of the wet to dry weights was calculated and used as a marker of fecal water content according to the following equation:

$$Water\;content\left(\%\right)=\frac{wet\;weight-dry\;weight}{wet\;weight}\times 100$$

#### 2.10.4. Disease Activity Index (DAI)

According to Murthy et al. [35], the disease activity index (DAI) was calculated as follows: total score (body weight loss + stool consistency + rectal hemorrhage)/3. Scores were given based on the percentage of weight loss (none = 0; 1–5% = 1; 5–10% = 2; 10–15% = 3; >15% = 4), stool consistency (normal = 0; pasty stool that does not stick to the anus = 1; pasty stool that does not stick to the anus = 2; pasty stool that stuck to the anus = 3; watery stool = 4), and rectal bleeding (hemoccult (-) = 0; hemoccult (±) = 1; hemoccult (+) = 2; hemoccult (++) = 3; obvious blood in stool = 4).

### 2.11. Statistical Analysis

The mean and standard error (SEM) of the mean were used to express the results. One-way analysis of variance (ANOVA) was used to assess the statistical variances among the various treatment groups and the post hoc Tukey’s test was used to confirm the results. Statistics were deemed significant at *p* ˂ 0.05. The GraphPad Prism application (version 4) was used to perform statistical analysis (GraphPad Software, San Diego, CA, USA).

## 3. Results

### 3.1. Box–Behnken Design (BBD) Optimization

By using the Box–Behnken Design (BBD), the current study improved the development of eluxadoline-loaded eudragit nanoparticles (ENPs) using the emulsion solvent diffusion method with varying proportions of eudragit (polymer) and polyvinyl alcohol (stabilizer). The particle size, PDI, and zeta potential (ZP) of the developed ENPs were examined as dependent factors in order to achieve the optimized formulation by BBD; these characteristics are shown in Table 2. The equation in terms of coded factors can be used to make predictions regarding the response for the given levels of each factor. The coded equation is useful for identifying the relative impact of the factors by comparing the factor coefficients [36]. The quadratic model was used to examine all dependent variables and 3D surface plots of the results were produced. The statistical analysis of the quadratic model discovered significant *p*-values, indicating a best fit for chosen responses (Table 3).

#### 3.1.1. The Effect of Independent Variables on Response Particle Size (Y1)

The selected independent factors affected the size of developed ENPs; the average size of several batches of formulations ranged from 286 to 634 nm, as shown in Table 2. The mathematical relationship between independent variables and particle size of developed ENPs is seen in the following polynomial Equation (1), that can be used to determine how factors and particle size are correlated using the coded equation.

Y1 = + 327.00 − 21.25 A − 41.12 B − 108.88C − 30.75 AB + 12.75 AC − 21.00 BC + 73.75 A^2^ + 106.00 B^2^ + 48.00 C^2^ (1)

According to response Y1, the above equation reflects the quantitative effects of independent variables (A, B, and C) and their interactions in terms of AB, AC, and BC. In this case A, B, C, AB, A^2^, B^2^, and C^2^ are significant (*p* ˂ 0.05) model terms. The model’s model F-value of 53.51 (*p* ˂ 0.05) proved that it was significant. A negative impact is indicated by the minus sign of the coefficient (A, B, and C), showing that the particle size decreased with the decrease in the concentration of EG, PVA, and sonication time. The predicted R^2^ of 0.7708 is in reasonable agreement with the adjusted R^2^ of 0.9673, i.e., the difference is less than 0.2 (Table 1). Adequate precision measures the signal to noise ratio. A ratio greater than 4 is desirable. The adequate precision of 18.90 indicates an adequate signal. This model can be used to navigate the design space.

**Figure 2:**
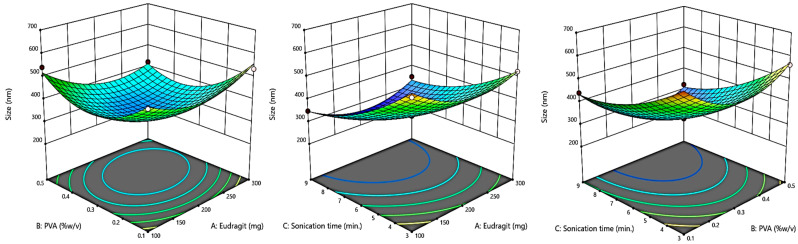
A 3D plot showing the effect of independent variables on particle size.

With the use of a 3D-response surface graph, interaction patterns were examined. It is clear from the 3D-response surface graph in Figure 2 that the concentrations of PVA and sonication time had a substantial impact on the particle size of ENPs. The particle size decreased with the increase in PVA concentration and sonication time.

#### 3.1.2. The Effect of Independent Variables on Response PDI (Y2)

**Figure 3:**
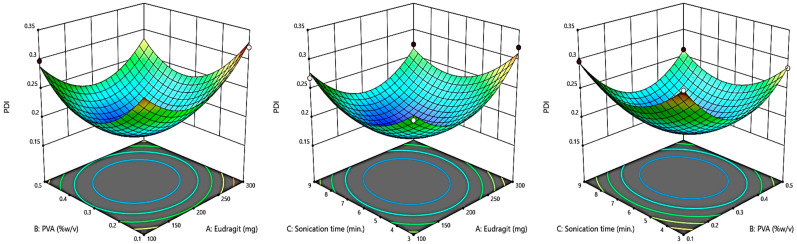
A 3D plot showing the effect of independent variables on PDI.

Table 2 lists the influence of independent variables on the PDI of various formulations. The experimental setup demonstrated that the quadratic model was the one that suited the data the best. The model was clearly fitted, as evidenced by the F-value of 65.96. The predicted R^2^ of 0.8135 was in reasonable agreement with the adjusted R^2^ of 0.9734, i.e., the difference was less than 0.2. Adequate precision measures the signal to noise ratio. A ratio greater than 4 is desirable. The ratio of 21.612 indicated an adequate signal. This model can be used to navigate the design space. Figure 3 shows graphs of the 3D actual and expected values of PDI, illustrating how the independent factors affected the PDI and showing how closely the two variables were related.

Y2 = + 0.1760 + 0.0059 A − 0.0146 B − 0.0105 C − 0.0083 AB − 0.0020 AC + 0.0591 A^2^ + 0.0661 B^2^ + 0.0474 C^2^ (2)

#### 3.1.3. The Effect of Independent Variables on Response ZP (Y3)

**Figure 4:**
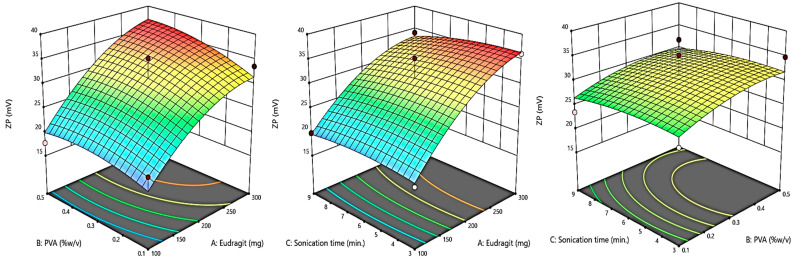
A 3D plot showing the effect of independent variables on zeta potential.

The surface charges of the ENP formulations with ELD loading ranged from 18.8 ± 3.84 to 36.1 ± 1.23 (Table 2). The presence of freely ionized amino groups, which are required for electrostatic repulsion between particles to generate stable nano-dispersions, was shown by the positive charge on the surface of the NPs. The mathematical representation of the measured response, Y3, as a polynomial equation is listed below. The stability of the developed formulations, where the nanoparticles have a tendency to de-aggregate rather than assemble, was significantly impacted by these high zeta potential values. The model F-value of 19.69 implied the model is significant. There was only a 0.04% chance that an F-value this large could occur due to noise. Figure 4 shows graphs of the 3D actual and expected values of zeta potential, illustrating how the independent factors affected the zeta potential.

Y3 = +31.20 + 6.84 A + 1.31 B + 0.6500 C − 0.0500 AB − 1.38 AC − 2.42 BC − 3.45 A^2^ − 0.1500 B^2^ + 0.0750 C^2^ (3)

For the analysis of particle size, PDI, and zeta potential, the obtained data suggested a quadratic model, which is shown in Table 3. The difference between adjusted and predicted R^2^ values for the investigated responses were less than 0.2, indicating a reasonable degree of agreement in the study design [37]. The particle size, PDI, and ZP of developed ENPs measured in the range of 286 to 634 nm, 0.263 to 0.321, and 18.8 to 36.1 mV, respectively (Table 2).

### 3.2. Selection of Optimized Formulation

**Figure 5:**
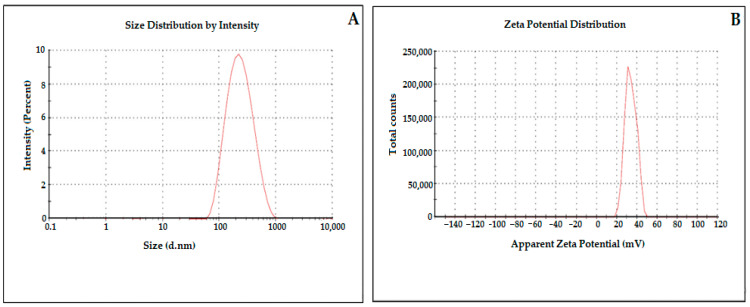
Particle size (**A**) and zeta potential (**B**) of optimized formulation (ENP2).

The ideal composition for the development of the optimized ELD-loaded EG-NP (ENP2) was identified by imposing constraints on the particle size, PDI, and ZP (Table 2); expert design software suggested an optimized composition with overall desirability 1.000. The software suggested an optimized formulation (ENP2) having a composition of eudragit (200 mg), PVA (0.5%, *w*/*v*), and sonication time (9 min.) The optimized formulation (ENP2) exhibited the particle size (286 ± 3.67 nm), PDI (0.263 ± 0.01) (Figure 5), and ZP (31.8 ± 3.18 mV) (Table 2).

### 3.3. Measurement of Particle Size, PDI, and Zeta Potential

Polymeric NPs must have a small particle size, low PDI, and high ZP values for the effective biodistribution of a drug [38]. The prepared ELD-loaded EG-NPs (ENP1-ENP14) were found to have a mean particle size in the range of 286–634 nm, PDI values between 0.176 and 0.321, and ZP between 18.8 and 36.1 mV. Based on particle characterization, ELD-loaded EG-NPs (ENP2) were optimized with particle size (286 ± 3.67 nm), PDI (0.263 ± 0.01) (Figure 5), and ZP (37.7 ± 3.18) (Table 2). The homogeneity of the particles in a nanocarrier system is shown by the PDI. Therefore, a good nanocarrier system should have a low PDI to deliver the drug and to increase oral bioavailability. The particles were considered extremely homogeneous, with a PDI value of 0.3 [39,40]. High zeta potential values were determined for each formulation, demonstrating the produced NPs having high stability. The ammonium quaternary groups of the eudragit, as was previously mentioned, caused the surface of the NPs to be positively charged [41,42]. The positively charged nanocarrier was typically advantageous due to its interactions with the negatively charged mucosal membrane. The drug that was encapsulated showed the prolonged residence time due to electrostatic interaction with the mucosal membrane in the small intestine [43].

### 3.4. Drug Encapsulation

The stability of the polymer matrix and the way the drug interacts with it both have a role in drug entrapment. The %DE of ELD-loaded EG-NPs (ENP2) showed 82.35 ± 4.1%.

### 3.5. DSC Analysis

**Figure 6:**
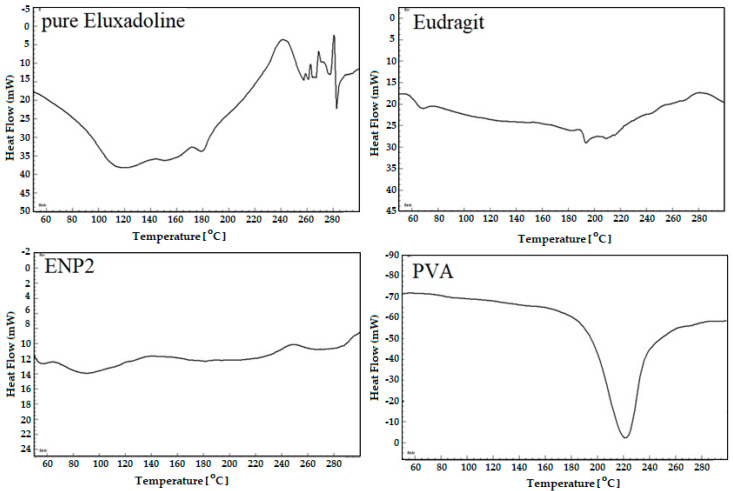
Comparative DSC spectra of pure ELD, eudragit, PVA, and optimized formulation (ENP2).

The DSC spectra of pure ELD, EG, PVA, and optimized ELD-loaded EG NPs (ENP2) were analyzed; the results are shown in Figure 6. The DSC spectra of ELD showed characteristic endothermic peaks at 118, 158, and 183 °C, as reported in the literature [42]. The DSC spectra of eudragit RS100 exhibited two endothermic peaks at 60 °C and 195 °C, related to water loss and melting temperature, respectively [44,45]. The DSC spectra of PVA showed melting endotherm at 221 °C [46]. The ELD peak completely disappeared from the spectra of the optimized ELD-loaded EG NPs (ENP2), probably due to encapsulation and stabilization of the drug by the polymer. The successful entrapment of ELD within the EG polymer was evidenced by the DSC studies.

### 3.6. FTIR Studies

**Figure 7:**
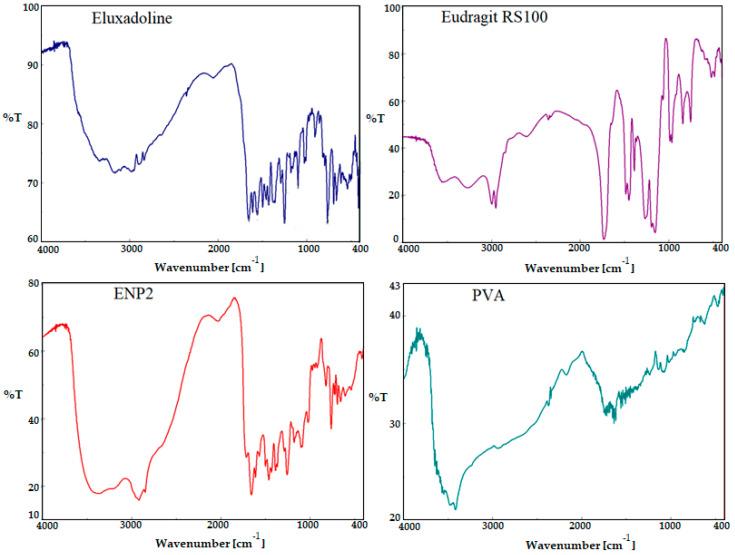
Comparative FTIR spectra of pure ELD, eudragit, PVA, and optimized formulation (ENP2).

A comparative FTIR spectra of pure ELD, EG, PVA, and optimized ELD-loaded EG NPs (ENP2) were studied for the identification of the encapsulated drug inside NPs (Figure 7). The pure ELD showed prominent functional group peaks at 3117 cm^−1^ (-NH- str), 3117 cm^−1^ (-NH-), 1696 cm^−1^ (-COOH), 1658 cm^−1^ (-RCONH2-), and 1429 cm^−1^ (-C=C-), which confirmed the purity of the drug [47]. The peaks at 3532 cm^−1^ and 3239 cm^−1^ (CH aliphatic stretching) were visible in the spectra of eudragit RS100 [48]. The characteristic absorption bands of ELD diminished or disappeared in the fingerprint region of the drug, which indicated that the drug encapsulated inside the eudragit polymer, and the appearance of new peaks in the ENP2 corresponding eudragit and PVA confirmed successful entrapment.

### 3.7. In Vitro Drug Release Studies

**Figure 8:**
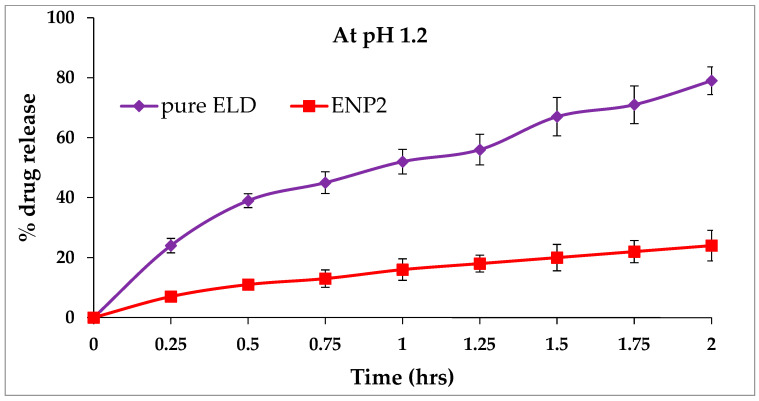
In vitro release profile of pure ELD and optimized formulation (ENP2) at pH 1.2.

**Figure 9:**
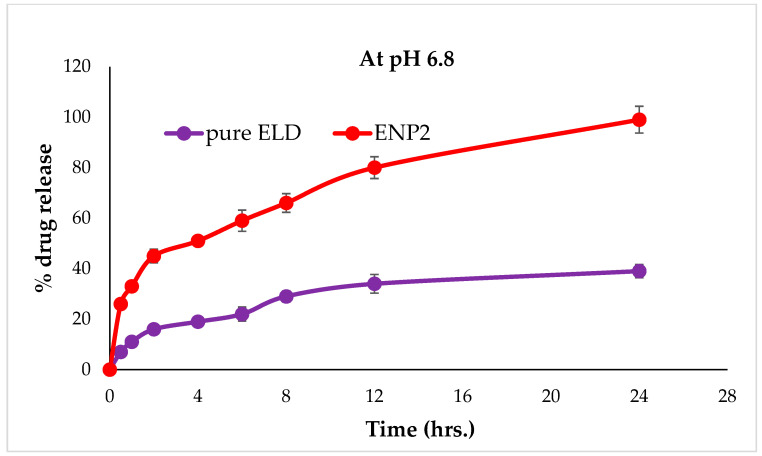
In vitro release profile of pure ELD and optimized formulation (ENP2) at pH 6.8.

Figure 8 and Figure 9, display the ELD release profiles at different pH levels. There was no drug adsorbed to the surface of the ENP2, as evidenced by the lack of burst releases of ELD in the beginning of the release profiles at pH 1.2 (Figure 8). However, at pH 6.8, a burst release could be seen at 2 h in the studies (Figure 9). The release profile of ELD from ENP2 at pH 1.2 exhibited a slow release in comparison with pure ELD, probably due to eudragit RS100 protection from the acidic environment. Over a 24 h period, the release of ELD from ENP2 in PBS (pH 6.8) was roughly 99% compared with less than 40% of pure ELD. These findings suggest that ELD was enclosed in eudragit RS100 polymeric nanoparticles and shielded from the stomach’s highly acidic environment. This indicates that the majority of ELD was released once the ENP2 reached the small intestine [49]. This nano-size of ENP2 enhanced drug release was due to the availability of a greater surface area (Figure 5). The release kinetics equations were used to study the ELD release pattern from ENP2 at two pHs (1.2 and 6.8). Applying the kinetic models, it was observed that the Higuchi model could adequately describe the release of ELD from ENP2 at both pHs (Table 3). The Higuchi model had the highest coefficient of correlation values among the applied release models (R2 = 0.8855 and 0.9680 at pH 1.2 and pH 6.8), as shown in Table 4. The best fit correlations were found by the Higuchi model based on the diffusion controlled release of the drug at both pHs [50].

### 3.8. SEM Studies

**Figure 10:**
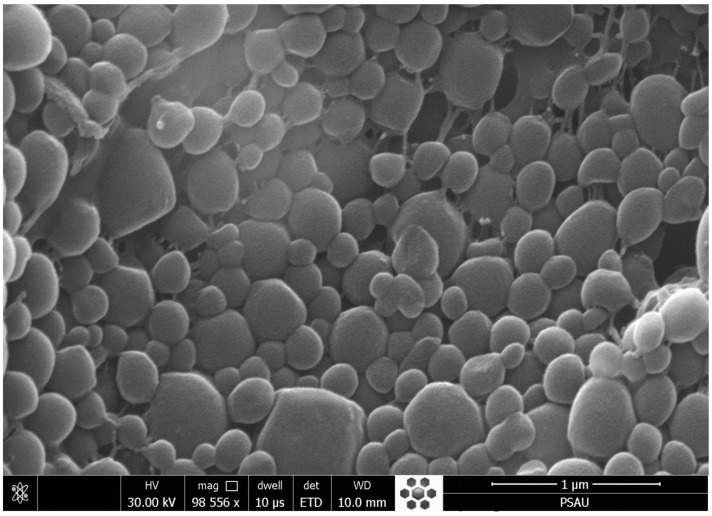
SEM images of optimized formulation (ENP2).

The SEM images of the optimized formulation (ENP2) demonstrated the smooth and spherical structure of particles (Figure 10). The particles were aggregated or stuck to each other, probably due to the PVA used in the formulation.

### 3.9. Assessment of Chronic Restraint Stress-Induced Irritable Bowel Syndrome

**Figure 11:**
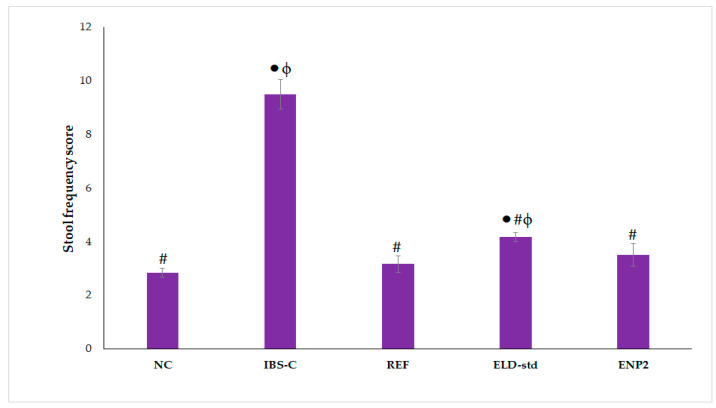
Effects of ELD-std and ENP2 on stool frequency in chronic restraint stress-induced IBS rats. Values are expressed as mean ± S.E.M., n = 6 rats/group. ● indicates significant difference versus normal control (NC) group at *p* < 0.05. # indicates significant difference versus IBS control (IBS-C) group at *p* < 0.05. ϕ indicates significant difference versus reference (REF) group at *p* < 0.05.

**Figure 12:**
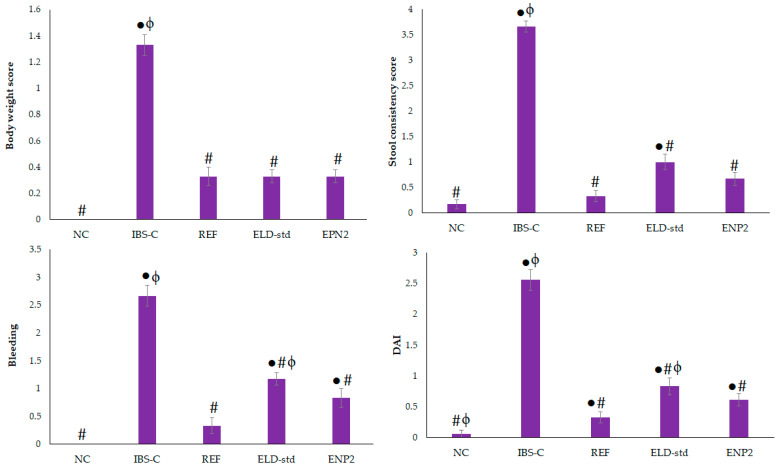
Effects of ELD-std and ENP2 on body weight, stool consistency, bleeding, and DAI scores in chronic restraint stress-induced IBS rats. Values are expressed as mean ± S.E.M., n = 6 rats/group. ● indicates significant difference versus normal control (NC) group at *p* < 0.05. # indicates significant difference versus IBS control (IBS-C) group at *p* < 0.05. ϕ indicates significant difference versus reference (REF) group at *p* < 0.05.

There were no observable behavioral or activity changes in the rats during the experiment and no illness or deaths from the therapy took place. According to Figure 11, IBS-C-induced rats had considerably more bowel movements than normal rats (*p* ˂ 0.05) and ENP2 dramatically decreased IBD-S rats’ bowel movements (*p* ˂ 0.05). The body weight of rats in the ENP2-treated group was significantly higher (*p* ˂ 0.05) than the ELD-std model group rats. Physical stool hardness reflects physical stool consistency, since it closely correlates with physical stool water content. A significant decrease in fecal water content in the ENP2 (45.47 ± 1.19%) -treated rats was observed compared with the ELD-std (53.36 ± 1.81%) and IBS-C (65.13 ± 2.29%) model group rats (Table 5 and Table 6). This finding suggests that chronic restraint stress (CRS) caused the stress models’ stools to include more water and to develop diarrhea. The formulation (ENP2) also exhibited reduced body weight, stool consistency, and bleeding scores in comparison with the ELD-std and control (IBS-C) groups (*p* < 0.05) (Figure 12). Thus, low scores of DAI of the formulation (ENP2) indicated a reduction in the severity of irritable-induced diarrhea. This improvement might be due to nano-sized particles and hence enhanced bioavailability. The results demonstrated that the formulation (ENP2) ameliorated IBS-D better than the pure ELD.

## 4. Conclusions

In this research work, ELD-loaded EG-NPs (ENP1-ENP14) were prepared using an eudragit polymer and PVA and formulations were optimized using a Box–Behnken Design software. The optimized ELD-loaded EG-NPs (ENP2) exhibited particle size, PDI, ZP, and drug entrapment 286 ± 3.67 nm, 0.263 ± 0.01, 31.8 ± 3.18 mV, and 82.35 ± 4.1%., respectively. The ENP2 exhibited enhanced and prolonged drug release at pH 6.8 due to the acidic protection of the drug by the eudragit RS100 polymer. The assessment of chronic restraint stress-induced irritable bowel syndrome revealed that ENP2 exhibited reduced body weight, stool consistency, and bleeding scores in comparison with the ELD-std and control (IBS-C) group (*p* < 0.05) and low scores of DAI of the formulation (ENP2) indicated a reduction in the severity of irritable-induced diarrhea. The formulation (ENP2) could be considered an effective treatment of IBS-D and other GIT-allied conditions. This novel drug delivery system needs to be further evaluated to ascertain its effectiveness and safety in in vivo experimental models before going on to the clinical stage.

## Figures and Tables

**Table 1:** Variables for the development of formulations.

Independent Variables	Levels		
	Low (−1)	Medium (0)	High (+1)
X1 = Eudragit (mg)	100	200	300
X2 = PVA (%*w*/*v*)	0.1	0.3	0.5
X3 = Sonication time (min.)	3	6	9
Responses	Target to		
Y1 = Size (nm)	Minimum size		
Y2 = PDI	Minimum PDI		
Y3 = Zeta potential	Maximum zeta potential		

**Table 2:** Developed formulations and observed responses.

Formulae (ENPs)	Eudragit (mg)	PVA (%*w*/*v*)	Sonication Time (min.)	Particle Size (nm)	PDI	ZP (mV)
ENP1	300	0.3	3	523 ± 5.43	0.321 ± 0.05	36.1 ± 1.23
ENP2	200	0.5	9	286 ± 3.67	0.263 ± 0.01	31.8 ± 3.18
ENP3	300	0.1	6	534 ± 8.24	0.321 ± 0.06	33.6 ± 4.60
ENP4	300	0.5	6	421 ± 6.55	0.276 ± 0.02	34.4 ± 2.94
ENP5	200	0.3	6	327 ± 4.83	0.176 ± 0.09	30.2 ± 3.21
ENP6	100	0.1	6	531 ± 6.51	0.31 ± 0.02	20.7 ± 5.27
ENP7	200	0.3	6	327 ± 2.36	0.176 ± 0.09	35.2 ± 3.21
ENP8	200	0.5	3	563 ± 4.57	0.286 ± 0.12	34.8 ± 4.44
ENP9	100	0.3	3	575 ± 9.41	0.267 ± 0.11	18.8 ± 3.84
ENP10	300	0.3	9	348 ± 5.25	0.273 ± 0.03	34.1 ± 3.21
ENP11	200	0.1	3	634 ± 3.69	0.312 ± 0.12	25.6 ± 6.52
ENP12	100	0.5	6	541 ± 8.37	0.298 ± 0.16	21.7 ± 6.31
ENP13	100	0.3	9	349 ± 6.95	0.269 ± 0.06	22.3 ± 5.48
ENP14	200	0.1	9	441 ± 8.89	0.297 ± 0.07	32.3 ± 2.63

**Table 3:** ANOVA data of the quadratic models.

Responses (Y)	R^2^	Adjusted R^2^	Predicted R^2^	Adequate Precision	SD	%CV	*p* Value
Y1	0.9857	0.9673	0.7708	18.90	20.70	4.70	Significant
Y2	0.9983	0.9734	0.8135	21.61	0.0093	3.61	Significant
Y3	0.9439	0.8719	0.6793	10.89	2.00	6.78	Significant

**Table 4:** Release kinetic models of EPN2.

Models	Equations	R^2^ (At pH 1.2)	R^2^ (At pH 6.8)
Zero order	Q = kt + b	0.6303	0.7943
First order	In(100 − Q) = −kt + b	0.6653	0.9550
Korsmeyer–Peppas	Q_t_/Q = k^n^	0.4626	0.3776
Higuchi	Q = kt^0.5^ + b	0.8855	0.9680

**Table 5:** Effects of ELD-std and formulation (ENP2) on body weight of IBD-S rats.

Groups	Body Weight		
	Day 0	Day 14	% wt Change
NC	116.33 ± 0.88	127.33 ± 2.23 #	9.42 ± 1.10 #
IBS-C	115.00 ± 0.93	111.33 ± 1.43 ●	−3.19 ± 1.02 ●
REF	116.33 ± 0.80	124.00 ± 3.07 #	6.59 ± 2.48 #
ELD-Std	115.67 ± 1.09	120.67 ± 1.71 ●#	4.39 ± 2.00 ●#
ENP2	115.83 ± 0.87	122.17 ± 3.59 #	5.44 ± 2.78 #

● indicates significant difference versus normal control (NC) group at *p* < 0.05. # indicates significant difference versus IBS control (IBS-C) group at *p* < 0.05.

**Table 6:** Effects of ELD-std and formulation (ENP2) on fecal weight and the percentage of water fecal content of IBD-S rats.

Groups	Fecal Weight (mg/h)		Fecal Water Content (%)
	Wet Weight	Dry Weight	
NC	314.50 ± 19.35 #	193.17 ± 19.69	39.25 ± 2.89 #
IBS-C	414.17 ± 16.03 ●	146.00 ± 14.77	65.13 ± 2.29 ●
REF	318.67 ± 13.37 #	184.50 ± 19.57	42.40 ± 4.59 #
ELD-Std	364.83 ± 9.20 ●#ϕ	169.83 ± 6.50	53.36 ± 1.81 ●#ϕ
ENP2	327.17 ± 16.51 #	177.83 ± 7.81	45.47 ± 1.19 #

Values are expressed as mean ± S.E.M., n = 6 rats/group. ● indicates significant difference versus normal control (NC) group at *p* < 0.05. # indicates significant difference versus IBS control (IBS-C) group at *p* < 0.05. ϕ indicates significant difference versus reference (REF) group at *p* < 0.05.

## Data Availability

The data presented in this study are available on request from the corresponding author.
